# Job Insecurity and Employees’ Extra-Role Behavior: Moderated Mediation Model of Negative Emotion and Workplace Friendship

**DOI:** 10.3389/fpsyg.2021.631062

**Published:** 2021-04-06

**Authors:** Shengxian Yu, Na Wu, Shanshi Liu, Xiaoxiao Gong

**Affiliations:** ^1^School of Business Administration, South China University of Technology, Guangzhou, China; ^2^School of Business Administration, Zhongnan University of Economics and Law, Wuhan, China; ^3^School of Business Administration, Southwestern University of Finance and Economics, Chengdu, China

**Keywords:** job insecurity, workplace friendship, extra-role behaviors, negative emotions, moderated mediation model

## Abstract

Based on the affective events theory, this paper discusses the influence of job insecurity on employees’ extra-role behavior. The mediating effect of negative emotion and the moderating effect of workplace friendship are also tested. The results of an empirical analysis, based on the data of 327 employees, show that job insecurity has a significant negative impact on employees’ extra-role behavior. Negative emotion plays a mediating role in the relationship between job insecurity and extra-role behavior. Workplace friendship moderated the relationship between job insecurity and negative emotions, as well as between job insecurity and extra-role behavior. Workplace friendship also moderates the mediating effect of negative emotion on the relationship between job insecurity and extra-role behavior, that is, the higher the level of the workplace friendship is, the weaker the mediation role mentioned above will be. The research results have implications for the sustainable development of the organization.

## Introduction

Under the background of dynamic competition and the rapid development of the platform economy, the employment environment has undergone profound changes. On the one hand, flexible employment is gradually replacing the traditional employment mode of “a job for life.” On the other hand, the flexible management modes in enterprises, like business outsourcing, crowd-sourcing, employee sharing, and other emerging employment modes, all serve to reduce workers’ security to some extent (Feng et al., 2008; Morteza, 2019). In the digital economy era, “temporary,” “flexible,” and “insecure” are more regarded as the labels of modern labor positions. The stability and predictability of employment have also been gradually replaced by job insecurity, which has become one of the most common workplace psychological problems faced by employees (Hu and Zhong, 2015; Kieun and Ki, 2019). In addition, the ravage of Covid-19 has a serious negative impact on the global economy and employment in 2019 (Wang et al., 2020b). As enterprises shut down and go bankrupt, employees are faced with problems such as job waiting, layoffs, and salary reduction, which greatly aggravates the job insecurity of employees. Job insecurity refers to an individual’s perception of the degree of uncertainty in the continuity of work (Greenhalgh and Rosenblatt, 1984). These perceptions include the fear of losing the job itself and the threat of losing the job’s benefits and characteristics (such as salary, position, etc.; Greenhalgh and Rosenblatt, 1984; De Witte, 1999). At present, there is much controversy about the resulting effects of job insecurity. Based on the traditional perspective of organizational behavior, most scholars believe that job insecurity is a kind of hindrance event or situation, which advocates eliminating employee job insecurity at work (Wang et al., 2019). They point out that job insecurity can have some negative effects, such as physical and mental health, life happiness, work attitude and behavior, etc. (Cheng and Chan, 2008; Zhou and Rong, 2011; Hur and Perry, 2020; Jung et al., 2021). A small number of researchers from the perspective of positive organizational behavior disagree with this view. Their studies indicate that job insecurity is not always harmful, for example, eliminating job insecurity leads to a “carefree” work environment and a “stagnant” work environment, reducing employees’ motivation for creativity and innovative behavior (Wong et al., 2005; Hu et al., 2014). On the contrary, the pressure caused by job insecurity will stimulate employees’ motivation to work. In order to keep their jobs, employees will work harder to deal with the threat of losing their jobs, thereby promoting higher task performance and more organizational citizenship behavior (Feather and Rauter, 2004; Wong et al., 2005). This controversy is enough to show that job insecurity has a differentiated impact on different situations, and with the quietly popularization of the “996” working system, it also indicates that it is increasingly difficult for organizations to eliminate the insecurity in work and provide employees with a “carefree” working environment. Therefore, future studies need to analyze specific issues. Based on this, in the context of Chinese organizations, studying the impact of job insecurity is of great significance to ensure the sustainable development of employees.

Extra-role behavior is widely concerned with job performance structure. The variable describes an employee’s spontaneous behavior that goes beyond the organization’s requirements and benefits the organization (Dyne et al., 1995). There are two reasons for choosing extra-role behavior as the outcome variable. First, there is a close relationship between extra-role behavior and job insecurity. Previous studies have shown that job insecurity can affect employees’ spontaneous and proactive behaviors (Dyne et al., 1995). Extra-role behavior can also precisely reflect the state of employees’ spontaneous behaviors. Second, the extra-role behavior of employees is related to the survival and development of enterprises. Innovation and voice behavior, the 996 working system, the spontaneous extension of working hours and other extra-role behaviors are all expected by the enterprise, as these behaviors play an important role in improving the competitiveness of the organization. Therefore, this study attempts to analyze the mechanism of job insecurity and extra-role behavior. Next, negative emotion is used as mediating variables. Emotion is usually regarded as a “by-product” of work (Jiang and Lavaysse, 2018), and related studies have also proposed that individual behavior is not only the result of rational processing, but also may be caused by emotion (Ashforth and Humphrey, 1995). Affective theory events also believe that emotion is the bridge connecting the characteristics of work environment and employee behavior (Weiss and Cropanzano, 1996). As a typical stress response, negative emotion is the state of fatigue after individuals perceive pressure in the workplace, which in turn often leads to employees’ negative reactions to others and a negative evaluation of themselves (Maslach et al., 2001). Compared with positive emotion, negative emotion is more likely to occur in organizations. According to the stressor-emotion framework, previous studies have focused on exploring the influence of different types of stressors on negative emotion, such as work pressure, leadership behavior, family conflicts, etc. (Maslach et al., 2001). However, these studies ignored the effect of uncertain and uncontrollable stressors, such as the impact of job insecurity on negative emotion. According to the emotional center model, negative emotion tends to reduce good behavior and increase bad behavior (Wei et al., 2019). Therefore, this study suggests that negative emotion is likely to play a mediating role between job insecurity and extra-role behavior. Finally, workplace friendship is introduced into the model as a moderating variable. One-third of the time is devoted to working in life, due to the necessary contacts and connections in work, individuals inevitably establish various complex relationship networks with others. Workplace friendship is the manifestation of interpersonal relationships in the workplace (Mao et al., 2012; Xiao et al., 2020). Its establishment and maintenance can promote employees’ mental health, provide employees with emotional support, and enhance employees’ job satisfaction and workplace happiness (Song, 2005). In addition, the studies have also pointed out that workplace friendship can positively affect employees’ attitudes toward work (Song and Olshfski, 2008; Yin et al., 2018). When employees experience job insecurity, it is necessary to further study whether the companionship and help among employees can help to alleviate negative emotion, and thus reduce the negative impact on extra-role behavior, it needs to be further studied. Therefore, in the workplace where there is job insecurity, taking workplace friendship as a research object to promote the release of negative emotion and relieve work pressure, it is in line with the actual situation of the organization and the demand of the theoretical model.

Based on the above analysis, this study mainly focuses on three points. First, analyze the relationship between job insecurity and off-role behavior. Second, according to the affective events theory (Weiss and Cropanzano, 1996), whether negative emotion have a mediating effect on the relationship between job insecurity and extra-role behavior; and third, under the condition of workplace friendship, can emotional and material satisfaction from interpersonal communication reduce negative emotion and thus change the mediating effect of negative emotions on the relationship between job insecurity and extra-role behavior? Thus changing the influence path of job insecurity, negative emotion, and extra-role behavior? This study takes large enterprises and institutions as samples, and expands the research on job insecurity by constructing and verifying relevant hypothesis models.

### Job Insecurity and Extra-Role Behavior

Job insecurity is treated as the perceived powerlessness to maintain desired continuity in a threatened job situation (De Witte, 1999). As a source of stress, job insecurity comes from two aspects, one is the perception of the risk of losing the current job. The other is the worry of losing or changing the job characteristics (salary, position, etc.; Rosenblatt and Greenhalgh, 2010; Jesus et al., 2019). Existing research conclusions on the outcome effects of job insecurity can be divided into two categories. First, it is believed that job insecurity, as a source of obstructive stress, has negative effects on job engagement, organizational commitment, happiness, etc. (Zhou and Rong, 2011; Hu and Zhong, 2015; Hur and Perry, 2020). The second suggests that job insecurity can be seen as a source of challenging stress, which has a positive effect on individual experience. For example, job insecurity can stimulate employees’ enthusiasm and creativity and improve their work performance (Wong et al., 2005). Therefore, scholars’ conclusions on the effects of job insecurity are still controversial, and as such, the impact of job insecurity should be analyzed on a case-by-case basis.

The influence of job insecurity on extra-role behavior can be illustrated from three aspects. First of all, job insecurity will cause individual fear, anxiety, and even depression, increasing the conflicts between the two sides of employment and thus reducing extra-role behavior. When an individual perceives a work-related threat and cannot deal with it, the individual will tend to reduce the resource investment of extra-role behavior and increase the resource investment of protecting himself/herself for reasons of self-protection and counterattack (Wang et al., 2015; Jesus et al., 2019). For example, empirical studies show that job insecurity violates autonomy, ability, and related basic needs. This leads to conflicts between employers and employees, which in turn greatly weakens employees’ perception of the working environment and thus reduces participants’ ability to solve problems creatively (Gilboa et al., 2008; Wang, 2014). Secondly, job insecurity will reduce an individual’s evaluation of job control, thus reducing the extra-role behavior. Whether job insecurity can be eliminated depends on the employee’s evaluation of work control. When an individual’s resources cannot eliminate the sense of insecurity or their evaluation of control is low, in order to solve the sense of insecurity caused by the stressors, an individual will usually reduce the resource investment in work, including extra-role behavior, as a means to express his resistance. Sverke and Hellgren (2001) compared and studied the relationship between job insecurity and the different responses of withdrawal, voice behavior, and loyalty behavior of unionized and non-unionized employees in a Swedish health center. The results showed that unionized employees were less likely to adopt the behavioral responses of withdrawal and voice behavior. They also showed more loyalty than non-unionized employees (Kieun and Ki, 2019; Hur and Perry, 2020). Finally, job insecurity will reduce an individual’s sense of belonging to the organization, thus reducing extra-role behavior. Through a questionnaire survey of 787 employees, researchers found that job insecurity and excessive competition insecurity have a significant negative correlation with organizational citizenship behavior (Hur et al., 2014). Based on this, the research proposes the following hypothesis,

*Hypothesis 1*: There will be a negative relationship between job insecurity and extra-role behavior.

### The Mediating Effect of Negative Emotion

According to the difference of valence, emotion can be divided into the categories of positive and negative. This study focuses on a negative emotion, which is generated by an individual in a specific behavior due to external or internal factors, such emotions are often accompanied by anxiety, anger, disgust, fear, etc., which are not conducive to normal thinking and task completion (Brief and Weiss, 2002; Farooq et al., 2017). For a long time, emotion has been seen as one of the factors that affect individual behavior. Negative emotion is often associated with avoidance motivation and is considered to be a means of avoiding actions and avoiding punishment. The affective events theory also points out that all kinds of events experienced by individuals at work can trigger individual emotional reactions. In addition, negative emotional experiences will lead to a negative understanding of the events or environment experienced by employees, thus resulting in a negative attitude (Weiss and Cropanzano, 1996).

In a workplace environment, job stressors often trigger both job insecurity and emotional response. According to the affective events theory (Weiss and Cropanzano, 1996), as a stressor, job identity insecurity will initially trigger the sufferer’s negative emotional experience. Studies have pointed out that job insecurity can create group division. Some individuals or groups of similar identity to other groups or work environments may express their distaste and think that their circumstances are worse than those in other groups, this will cause the individuals to have negative feelings and emotion (Lai et al., 2013; Wang et al., 2020b). It can be said that job insecurity makes it difficult to meet the demands related to employees’ positive self-esteem. This is especially true when social concepts limit the employee’s possibility of building a positive self, and hence, they will experience even more negative emotion. Similarly, Zhang et al. (2013) believed that job insecurity not only induces negative emotion (such as depression and pain) but also leads to the exhaustion of employees’ psychological resources. In addition, job insecurity caused by the working environment will make employees feel nervous and uneasy, thereby reducing their sense of belonging and loyalty to the organization.

*Hypothesis 2*: There will be a positive relationship between job insecurity and negative emotion.

Emotions have a certain predictive effect on certain behaviors, especially negative emotion. These behaviors include flight caused by fear, attacks caused by anger, expulsion caused by disgust, etc. (Guo and Wang, 2007). A negative emotional state in employees will affect their work attitude and thoughts, thus affecting role behaviors (Wei et al., 2019). Therefore, this study believes that negative emotion will have an impact on extra-role behavior, which can be explained by the affective events theory. The affective events theory points out that emotion is the bridge connecting the characteristics of the work environment with the behavior of employees (Weiss and Cropanzano, 1996). That is to say, the work events experienced by employees will first trigger emotional reactions and then affect their behaviors through emotional reaction (Weiss and Cropanzano, 1996). Since it was first proposed, the affective events theory has been widely used to explain the “black box” of the working environment’s action mechanism on employees’ behavior. Borman et al. (2001) found that a significant relationship exists between concentration, anxiety, and anger mediating stressors, and organizational citizenship behavior and counterproductive behavior. Researchers conducting a mood consistency theory angle of view analysis found that, with negative emotion and altruistic behavior, a significant negative correlation exists between organizational citizenship behavior and so on. These researchers maintained that employee behavior will be affected by a bad mood. When an individual’s emotional state is bad, they will negatively evaluate their work affairs. This will result in employees forming a negative evaluation of the organization and their colleagues (Yang et al., 2017; Wei et al., 2019). If such negative evaluations affect the behavioral decisions of employees, the motivation of employees to volunteer to help the organization and colleagues will be reduced. In addition, negative emotion can cause employees to be more aggressive and less friendly at work, which can in turn adversely affect their interpersonal and work communications. In addition, some researchers have analyzed negative emotion from other perspectives. These studies have found that, even if employees have negative emotion in the workplace, they will disguise them and thus consume a lot of resources in controlling their negative emotion. This will lead to the loss of self-control resources and thus reduce extra-role behaviors (Hu and Zuo, 2007). As an important source of stress, job insecurity is bound to arouse negative emotion, such as anger, frustration, and pain, thus reducing employees’ extra-role behavior. Therefore, it can be inferred that negative emotion has a mediating effect on the relationship between job insecurity and extra-role behavior. Based on the above analysis, the following hypothesis is proposed.

*Hypothesis 3*: Negative emotion plays a mediating role in the associations between job insecurity and extra-role behavior.

### The Moderating Effect of Workplace Friendship

Workplace friendship is “a kind of non-coercive interpersonal relationship formed on the base on the voluntary principle” (Wright, 1984). As a variable of emotional connection, workplace friendship has a self-regulating effect and has been widely discussed in the fields of psychology and organizational behavior. To be specific, workplace friendship is a friendly relationship, with voluntary, non-exclusive, and personal characteristics, developed among colleagues based on formal work contact (Berman et al., 2002). A high level of workplace friendship will compel individuals to show a strong willingness to share benefits and resources (Anjum et al., 2019). Workplace friendship not only meets the individual’s emotional needs in the workplace, but also contributes to knowledge sharing and mutual help among colleagues. This makes it easier to form a harmonious working atmosphere of mutual trust and love, and increases resource investment in extra-role behavior. According to the affective events theory (Weiss and Cropanzano, 1996), individual emotion influences cognitive and behavioral decisions. In addition, as an emotional connection, workplace friendship is bound to influence employees’ workplace emotion and behavior.

In fact, in this study, there are three reasons for the moderating effect of workplace friendship on the relationship between job insecurity and negative emotion. One is the relationship between workplace friendship and supportive social resources. Workplace friendship is a kind of conditional resource, which determines the potential of individuals or groups to work under pressure (Mao et al., 2012). In the case of friendships in higher vocational colleges, employees can obtain more support from the social resources of friendship. In addition, the support from the social resources of friendship can help individuals or groups to enhance their anti-pressure capital and confidence, thus helping them to maintain their state or quickly recover their emotional state in the face of job insecurity. Second, there is a relationship between workplace friendship and emotional performance. Workplace friendship has a positive effect on employees’ working attitudes (Berman et al., 2002). In other words, positive interpersonal relationships create a relaxed, enjoyable, and harmonious work environment, provide emotional support for employees, and mitigate the effects of job insecurity. In addition, a good workplace atmosphere is conducive to the sharing of knowledge and information, the improvement of skills, the enhancement of employability, and the elimination of the impact of job insecurity on oneself. Third, there is a relationship between workplace friendship and security. The experience of feeling safe, which is brought about by good workplace friendships and a good working atmosphere, will also cause employees to be more optimistic about their work tasks and reduce the uneasy caused by job insecurity. Based on the above analysis, the following hypothesis is proposed,

*Hypothesis 4*: Workplace friendship will moderate the relationship between job insecurity and negative emotion.

Introducing workplace friendship in the field of organizational behavior, according to the above discussion, negative emotion not only plays a mediating role in the relationship between job insecurity and extra-role behavior, but also the magnitude of the mediating role will be affected by the level of workplace friendship. Workplace friendship plays a negative moderating role in the action path of “job insecurity - negative emotion - extra-role behavior.” Accordingly, the following hypothesis is proposed.

*Hypothesis 5*: The indirect effect of job insecurity on extra-role behavior via negative emotion is moderated by workplace friendship, such that the indirect effect will be weakened for employees with high levels of workplace friendship.

**Figure 1:**
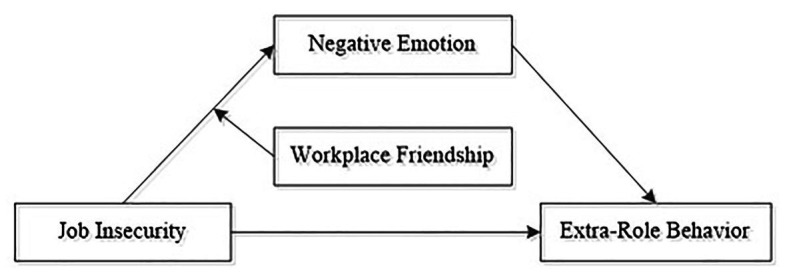
Theoretical framework of the study.

Altogether, we summarize our research variables and hypotheses in a conceptual framework in Figure 1.

## Materials and Methods

### Procedure and Participants

The sample of this research comes from four financial institutions in China, and the paper questionnaire is distributed with the cooperation of the organization’s human resources department. Before the formal survey, this study conducted a small sample pre-test, distributed 50 questionnaires, and recovered 42 valid questionnaires. Cronbach’s alpha and principal component analysis is used to verify the reliability and effectiveness of the scale. The analysis of 42 valid samples shows that the reliability values of the four scales involved are all greater than 0.7, and the factor loading values are all greater than 0.5. It shows that the scale has good reliability and validity. The questionnaire survey was conducted in two stages, 1 month apart. The specific situation is as follows: A total of 385 questionnaires were issued in the first survey, and 366 valid questionnaires were recovered, mainly measuring job insecurity, workplace friendship, and control variables. In the second survey, a total of 366 questionnaires were issued and 352 valid questionnaires were returned, mainly measuring employees’ negative emotions and extra-role behavior. After the two questionnaires were matched, 327 valid questionnaires were obtained, and the final effective response rate was 84.93%. Through the statistical analysis of the survey samples, it is found that in terms of sample gender, the proportion of men is 43.74%; the age is mainly 30–40 years old, accounting for 44.01%; in terms of education, a bachelor degree is the mainstay, accounting for 66.57%. The survey mainly focuses on frontline employees, accounting for 76.88%.

### Measures

In order to ensure the accuracy of the empirical analysis, this research scale refers to authoritative journal literature and selects mature scales with high reliability and validity. All scales are developed by foreign scholars, and the original scale language is English. The scale was translated and back-translated according to the procedure proposed by Brislin (1986). Three bilingual doctors were invited to translate and back-translate the scale, and two experts in the field of organizational behavior were invited to conduct a survey on the scale and adjust it according to the situation. All items use a 5-point Likert scale (1 = strongly disagree and 5 = completely agree) in the survey.

### Job Insecurity

The 7-item scale developed by Hellgren et al. (1999) was adopted. It includes two dimensions quantitative job insecurity and quality job insecurity, the quantitative job insecurity includes three items, typical questions such as “I am worried that I will be fired in the future” and “I feel uneasy that I may lose my job in the future.” The quality job insecurity includes four items, typical questions such as “I think the organization will make my work more challenging in the future.” The reliability and validity of the scale have been verified by many studies. Such as Hellgren et al. (1999) used this scale to verify the relationship between job insecurity and employee attitude, and the reliability reached 0.79, Chinese scholar Liu et al. (2019) applied this scale to test the context of innovation behavior, and the reliability reached 0.822. In this part of the study, the McDonald’s Omega and Coefficient Alpha are both 0.879.

### Negative Emotion

The 5-item scale developed by Liu et al. (2007) was adopted. This scale is mainly used to measure negative emotions with high arousal. In addition, negative emotions with high arousal usually have a more obvious impact on an individual’s experience, happiness, behavior, etc. Typical statements in the scale include “My work makes me angry.” Liu et al. (2007) tested the scale in China and abroad, and the reliability reached 0.89 and 0.86. In this part of the study, the McDonald’s Omega and Coefficient Alpha are 0.805 and 0.804, respectively.

### Extra-Role Behavior

The 4-item scale developed by Eisenberger et al. (2001) was used, which includes helping behavior, innovation behavior, and voice behavior in extra-role behaviors. Typical statements in this scale included “you usually help those who are absent to complete their work.” This scale has been recognized and widely used by many scholars (Liu et al., 2016; Wu et al., 2017). In this part of the study, the McDonald’s Omega and Coefficient Alpha are both 0.869.

### Workplace Friendship

The 6-item scale developed by Nielsen (2000) was used. The scale mainly measures individuals’ feelings about the friendliness of interpersonal relationships within the organization, which should be rich in mutual commitment, trust, and shared values and fun in work and life. Typical statements in this scale include “I have the opportunity to get to know my colleagues.” This scale has been recognized and widely used by many scholars (Liang, 2006; Qin, 2016). In this study, the McDonald’s Omega and Coefficient Alpha are 0.957 and 0.856, respectively.

### Control Variables

Control variables: In this study, the control variables include gender, marriage, age, education background, and position. The dummy variable of gender is processed, and the value of females is 0, and that of a male is 1. Marriage is divided into three levels: unmarried, married, and divorced. The age is divided into four grades: under 30 years old, 31–40 years old, 41–50 years old, and above 50 years old. The education level is divided into four levels: college degree and below, bachelor’s degree, master’s degree, and doctor’s degree. The positions are divided into four levels: staff, junior management, middle management, and senior management. The first part contained questions about the participants’ demographic information (e.g., age, gender, and education level) because these characteristics can play a significant role in predicting employees’ attitudes (Williams and Hazer, 1986). In addition, according to the research of domestic scholars Chen and Zhang, education background and position also influence behavior and cognition, which is also in line with the cultural background of high power distance.

## Results

### Confirmatory Factor Analysis

In this study, AMOS7.0 software was used to perform discriminative validity tests among constructs for job insecurity, workplace friendship, negative emotion, and extra-role behavior. The analysis results are shown in Table 1. The comparison showed that the four-factor model had the best fitting degree (*χ*^2^ = 446.07, *df* = 203, *χ*^2^/*df* = 2.197, RMSEA = 0.061, SRMR = 0.068, TLI = 0.937, GFI = 0.899, NFI = 0.945, CFI = 0.915). In addition to the four-factor benchmark model, this paper tests the three competitive models of the combination of variables, and their goodness of fit is weaker than the benchmark model, indicating that the five variables in this paper have good discriminant validity.

**Table 1:** Confirmatory factor analysis.

Variable	*χ*^2^	*df*	*χ*^2^/*df*	SRMR	RMSEA	TLI	GFI	NFI	CFI
Four-factor model (JI, NE, WF, EB)	446.07	203	2.197	0.068	0.061	0.937	0.899	0.903	0.945
Three-factor model (JI, NE+WF, EB)	933.87	206	4.533	0.161	0.104	0.814	0.754	0.798	0.834
Two-factor model (JI+NE+WF, EB)	1529.39	208	7.352	0.194	0.140	0.672	0.648	0.669	0.699
Single factor model (JI+NE+WF+EB)	2336.25	209	11.178	0.249	0.177	0.464	0.466	0.494	0.515

JI, job insecurity; NE, negative emotion; EB, extra-role behavior; WF, workplace friendship.

### Descriptive Statistics

Table 2 presents the descriptive statistics and correlations for the study variables. The descriptive statistics of the scales (means and SDs) and the Pearson correlations between the variables are reported in Table 2. As can be seen, a significant negative correlation exists between job insecurity and extra-role behaviors (*r* = −0.185, *p* < 0.01). Job insecurity was significantly positively correlated with negative emotions (*r* = 0.495, *p* < 0.01), and negative emotions were significantly negatively correlated with employees’ extra-role behaviors (*r* = −0.206, *p* < 0.01).

**Table 2:** Descriptive statistical results and correlation coefficients.

Variable	Mean	SD	Maximum	Minimum	1	2	3
Job insecurity	2.67	0.86	4.14	1.43			
Negative emotion	2.67	0.81	4.40	1.40	0.495^**^		
Extra-role behavior	2.79	0.94	4.75	1.25	−0.185^**^	−0.206^**^	
Workplace friendship	3.05	0.77	4.50	1.42	−0.178^**^	−0.118^*^	0.388^**^

* *p* < 0.05;

** *p* < 0.01;

*** *p* < 0.001.

### Hypothesis Test

#### Analysis of Main and Mediating Effects

This research uses SPSS24.0 software and the Process3.0 program developed by Hayes (2012) to examine the main effect of job insecurity on employees’ extra-role behavior and the mediating effect of negative emotion. The study sets age, marriage, gender, education, and position as control variables, with a confidence interval of 95%, and 5,000 Bootstrap samples were conducted. The results of the analysis are shown in Table 3. Job insecurity has a significant impact on employees’ extra-role behaviors (M1, *B* = −0.176, *p* < 0.01), that is, the higher the experience of job insecurity, the less likely to implement extra-role behavior. Hypothesis 1 is verified. Similarly, there is a positive correlation between job insecurity and negative emotion (M3, *B* = 0.474, *p* < 0.001), Hypothesis 2 is supported. It can be seen from Table 3, job insecurity is significantly related to negative emotion (*B* = 0.474, *p* < 0.001), and negative emotion is significantly related to extra-role behavior (*B* = −0.154, *p* < 0.05), hence, negative emotions have a significant mediating effect (*B* = 0.474 × −0.154 = −0.072, *p* < 0.05). Thus, Hypothesis 3 is supported.

**Table 3:** Mediating and moderating effects.

Variable	Extra-role behavior				Negative emotion			
	M1		M2		M3		M4	
	*B*	*SE*	*B*	*SE*	*B*	*SE*	*B*	*SE*
Sex	0.139	0.103	0.117	0.103	−0.143	0.078	−0.134	0.076
Marriage	−0.114	0.096	−0.091	0.096	0.150^*^	0.073	0.149^*^	0.078
Age	0.083	0.063	0.062	0.063	−0.139^***^	0.048	−0.150^**^	0.047
Position	−0.266^**^	0.091	−0.262^**^	0.090	0.027	0.069	0.071	0.068
Education	0.032	0.093	0.039	0.093	0.042	0.071	0.056	0.069
Job insecurity	−0.176^**^	0.059	−0.103	0.068	0.474^***^	0.045	0.208^***^	0.181
Negative emotion			−0.154^*^	0.073				
Workplace friendship							0.636^***^	0.164
Interactive items							−0.239^***^	0.057
F	4.182^***^		4.261^***^		20.579^***^		18.403^***^	
*R*^2^	0.072^***^		0.085^***^		0.278^***^		0.316^***^	

Interactive items = Job insecurity*Workplace friendship.

* *p* < 0.05;

** *p* < 0.01;

*** *p* < 0.001.

#### Moderating Effect Analysis

In this study, the variance inflation factor (VIF) of the four variables was tested before the moderating effect analysis, and the results were all less than 1.51, indicating that there was no multicollinearity problem among the variables. Then, the moderating effect of negative emotions was analyzed by the PROCESS macro program (see Table 3). Negative emotion is significantly related to job insecurity (M4, *B* = 0.474, *p* < 0.001) and workplace friendship (M4, *B* = 0.636, *p* < 0.001), and the interaction term coefficient between job insecurity and workplace friendship is also significant (M4, *B* = −0.239, *p* < 0.001), which shows that workplace friendship has a moderating effect between job insecurity and negative emotion. Hypothesis 4 is verified.

**Figure 2:**
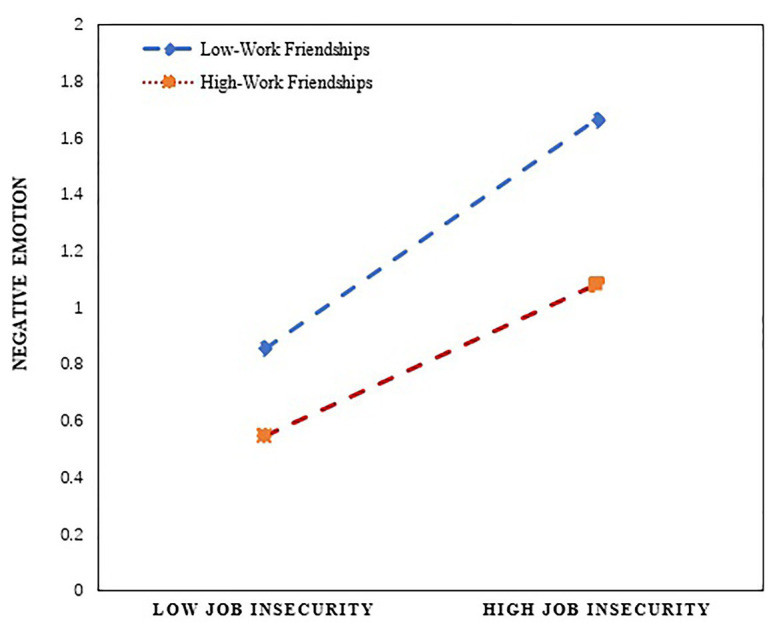
Moderating effect of workplace friendship between job insecurity and negative emotion.

In order to more vividly express and illustrate the moderating effect of workplace friendship, this study drew a pitch diagram of the relationship between job insecurity and employees’ negative emotions. The diagram is based on the drawing method and procedure proposed by Aiken and West (1991; see Figure 2). As can also be seen from Figure 2, workplace friendship does not change the positive relationship between job insecurity and negative emotions. However, workplace friendship can adjust and affect the relationship between employees. Employees with relatively low workplace friendship (M − 1SD) and employees with high vocational friendship (M + 1SD) can alleviate the positive effect of job insecurity on negative emotion. Hypothesis 4 is thus further supported. Finally, this study examines the moderating mediating effect. According to the suggestions of Edwards et al., the PROCESS macro program was used to conduct 5,000 repeated data sampling, in order to report the indirect effects and index indicators. The bootstrap method was used to obtain the conditional indirect effect under the condition of 1 SD, plus or minus the mean value of employees’ workplace friendship (see Table 4). The Table 4 data show that, under the low and high value in workplace friendship, job insecurity by way of negative emotion affects employee extra-role behavior the indirect effects, respectively, to 0.044 [CI (0.090, 0.007)] and 0.113 [CI (0.202, 0.021)]. The confidence interval does not contain 0, which shows that, under the influence of workplace friendship, job insecurity has a significant indirect effect on employees’ extra-role behavior through negative emotion. However, since the indirect effects of the moderating variables with both high and low values are significant, it is difficult to judge whether there is a moderating mediating effect based solely on the conditional indirect effect analysis. Therefore, it is necessary to further use the PROCESS program to obtain the significance evaluation index value of the moderating mediating effect. As can be seen from the data on the right side of Table 4, the index of the moderating effect of negative emotions on the indirect relationship between job insecurity and extra-role behavior is 0.282 [CI (0.004, 0.575)], and the confidence interval does not include 0. Therefore, workplace friendship has a moderating effect on the indirect effect of job insecurity and extra-role behavior. Thus, Hypothesis 5 is supported.

**Table 4:** Moderated mediating effects.

Moderate variance	Job insecurity→ Negative emotion→ Extra-role behavior							
	Indirect effect				Moderating mediating effect			
	EFFECT	SE	LLCI	ULCI	INDEX	SE	LLCI	ULCI
Low-WF	−0.044	0.021	−0.090	−0.007	0.282	0.0137	0.004	0.575
High-WF	−0.113	0.046	−0.202	−0.021				

**Figure 3:**
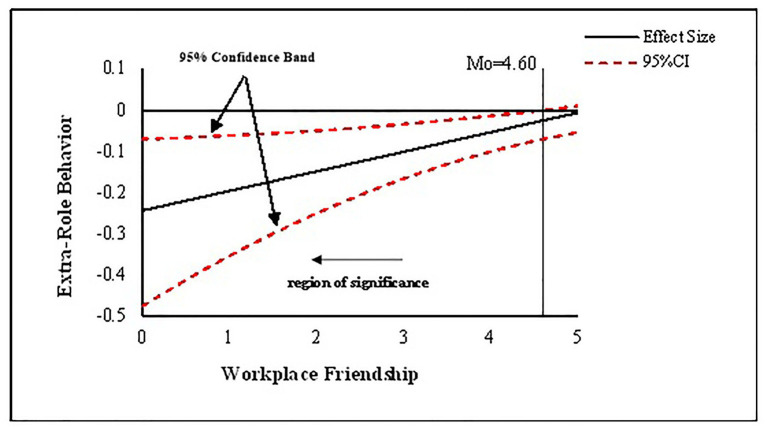
The moderating effect of workplace friendships on the indirect relationship of job insecurity with extra-role behavior through negative emotion.

As mentioned above, the moderated mediating effect involved in this study is a linear function of the moderating variable. However, previous testing methods (such as subgroup analysis and difference analysis) can only show the indirect effects under two different values of the moderators. These methods cannot fully reflect the full picture of the indirect effects affected by the moderators. In order to overcome this defect, this research referenced the recent practice of some scholars (Sun et al., 2014). This was done by running the Preacher and SPSS grammar program (Preacher et al., 2007), utilizing one’s own Johnson Neyman method (Preacher et al., 2007), and calculating the 95% confidence and significant domain-specific values in the form of a graphic. This more clearly shows the regulating continuous variable value under the condition of the indirect effect. The line in Figure 3 represents the moderated mediating effect on the three dependent variables. This is a linear function of the moderators, while the dotted line represents the corresponding confidence band. As can be seen from Figure 3, when the value of workplace friendship is less than 4.60 (full score = 5), job insecurity has a significant indirect effect on extra-role behavior through negative emotion, which further confirms the proposition in Hypothesis 5, which states that workplace friendship plays a moderating role in the mediation process of negative emotion.

## Discussion

In this study, 327 employees of financial companies were used as the research object. Through the mediating effect of negative emotion and the moderating effect of workplace friendship, this study explored the relationship between job insecurity and employees’ extra-role behavior. The results show that there is a negative relationship between job insecurity and extra-role behavior; negative emotion play a mediating role between job insecurity and extra-role behavior, workplace friendship has a moderating effect on the relationship between job insecurity and negative emotion.

The verification results of the relationship between job insecurity and extra-role behavior are consistent with previous research conclusions (Zhou and Rong, 2011), that is, job insecurity as a negative stressor will have a negative impact on extra-role behavior. On the one hand, when employees perceive that their job position or job characteristics are threatened, under the principle of reciprocity, employees will reduce their organizational loyalty and sense of belonging, which ultimately negatively affects behavior. According to the affective events theory (Weiss and Cropanzano, 1996), when employees perceive that their work is unstable or poor working conditions, they will have negative perceptions. Based on the principle of reciprocity, employees will correspondingly reduce the extra-role input of psychological, physical, and emotional resources. In addition, research also confirms the mediating role of negative emotions between job insecurity and extra-role behavior. Consistent with the research of Jiang and Lavaysse (2018), job insecurity has a negative impact on employees’ emotions. According to the affecting event theory, the situation of the event will have an impact on individual perception and behavior. When employees face the uncontrollability of the work situation and are unable to do anything, they will put themselves in a state of anxiety and fatigue, consume a lot of psychological and emotional resources, and it is difficult to carry out extra-role behavior.

Workplace friendship moderated the mediating effect between job insecurity and extra-role behavior. Job insecurity can lead to increased negative emotion, compared with low-level workplace friendship, employees with high-level workplace friendships show higher extra-role behavior. Similarly, Anjum et al. (2019) also point out that workplace friendship can reduce the impact of negative workplace events on employees, and even stimulate employees’ work enthusiasm and behavior. Specifically, job insecurity causes employees to lose their sense of belonging and loyalty to the organization, resulting in the emotional loss. If they cannot get effective support and emotional supplements from colleagues, they will lead to reduced extra-role behavior. However, workplace friendships can offset negative emotions and reduce the negative consequences of job insecurity. In summary, job insecurity will have an adverse effect on role behavior, and employee workplace friendships can significantly reduce this negative effect.

### Theoretical Contribution

This study makes the following theoretical contributions: (1) This study confirms that job insecurity is an important antecedent variable of employees’ extra-role behavior. Extra-role behavior is an active and spontaneous behavior of employees. Job insecurity has the same inhibitory effect on extra-role behavior, which is consistent with the research conclusion of scholars that job insecurity can reduce employees’ work initiative (De Beer et al., 2016; Sverke et al., 2019). This study further shows that job insecurity has a profound impact on employees’ psychology and behavior, and can inhibit employees’ work enthusiasm. Therefore, organizations should pay attention to the impact of job insecurity on employees’ psychology and behavior in the workplace. (2) This study found that negative emotions plays a mediating role between job insecurity and extra-role behavior. On the one hand, this conclusion explains the internal mechanism of job insecurity affecting employee extra-role behavior. The research conclusion is consistent with the previous study on the effect of job insecurity on behavior (Wong et al., 2005; Wang et al., 2020a), the research conclusion can be a useful supplement to the research on the effect of job insecurity. On the other hand, it once again supports the affective events theory in the Chinese context, that is, emotion is an intermediary factor connecting work situation events and individual behavior (Weiss and Cropanzano, 1996). When the work environment or characteristics make employees experience insecurities, it will prompt employees to have negative emotional perceptions, thereby affecting their resource investment in extra-role behavior. More importantly, compared to previous studies on job insecurity and extra-role behavior (Cheng and Chan, 2008), there is no research that integrates job insecurity, negative emotion, and extra-role behavior, this study puts forward and verifies that job insecurity affects employees’ extra-role behavior through the mediating effect of negative emotion, and theoretically proves the relationship between the three. (3) This study also found that workplace friendship significantly negatively moderating the relationship between job insecurity and negative emotion. The results verify that extra-role behavior is the result of interaction between situational factors and individual factors. Previous literature mainly studied workplace friendship as a pre-dependent variable or outcome variable (Potgieter et al., 2018; Xiao et al., 2020), and seldom analyzed the role of workplace friendship from the perspective of boundary conditions. In this study, the moderating role of workplace friendship between job insecurity and negative emotion was explored in the workplace context, which expanded the research perspective of workplace friendship. In addition, from the moderating effect diagram, it can be seen that compared with low workplace friendships, high workplace friendships can reduce the negative emotions generated by job insecurity. The research conclusions are beneficial to the application of organizational human resource management processes, and workplace friendships can improve employees’ work mood and positivity.

### Practical Implications

The affective events theory not only provides a theoretical model for studying the coping process mechanism of job insecurity but also provides an operational method to intervene in the problem of job insecurity. (1) This study found that job insecurity can negatively affect employees’ extra-role behavior. Enterprise managers can establish psychological counseling and training mechanisms to alleviate the negative impact of job insecurity on extra-role behavior. In addition, the enterprise should provide employees with valuable work characteristics, which can help to enhance the organizational loyalty and work enthusiasm of employees in the current unstable environment. (2) This study also found that negative emotion plays a mediating role, which requires the organization must pay attention to the change of employees’ emotion, does a good job of emotional guidance. On the one hand, managers should provide more work-related support, such as skill support, experience imparting, and salary incentive, to enhance employees’ confidence in alleviating job insecurity. On the other hand, when employees show their worries and anxiety about losing their jobs, psychological counseling and team building activities can alleviate the negative emotion of employees. (3) This study found that workplace friendship played a moderating role in the overall mechanism. This shows that managers should pay more attention to the maintenance of relationships between colleagues. On the one hand, the organization should establish an inclusive, harmonious, open, and communication platform, and create a good environment for the development of friendship among employees. On the other hand, the enterprise should recruit employees who are easy to get along with others, and maintain internal colleague interaction and interpersonal relationship.

### Strengths and Limitations

The conclusions of this study support the proposed hypotheses, but there are still some limitations in terms of data, theory, variables, and other aspects of the study. (1) From the perspective of research design, although the data were collected from two-time points, the mediating variable (negative emotion) and the outcome variable (extra-role behavior) were collected at the same time point in the research framework. Therefore, it is not conducive to infer the causal relationship between the variables. In future studies, a three-stage longitudinal follow-up survey or experiment should be attempted to further verify the relationship between variables (Spector, 2019; Ruhle et al., 2020). In addition, the sample data came from a single source, all variables are employee self-evaluation. Although the survey is conducted anonymously, it may also lead to data homology error, which would affect the research conclusion (Spector, 2006). In future research, a paired questionnaire can be filled out by supervisors and employees to improve the credibility of the results. (2) This study confirms that job insecurity has a negative effect on extra-role behavior. However, some scholars have proposed that job insecurity also plays a positive role (Feather and Rauter, 2004; Wong et al., 2005). This study only analyzes job insecurity from a negative perspective, in order to explore the influence of job insecurity on extra-role behaviors through an emotional state. In the future, the positive effects of such stressors on behavior could also be explored from various perspectives. In addition, this study only verifies employee insecurity at an individual level. Organizational and team levels need to be further explored, in order to better understand the mechanism of job insecurity. (3) This study only discusses the moderating effect of workplace friendship on the mechanism of job insecurity. In the real workplace, organization and team levels, such as work characteristics, leadership characteristics, team environment, and human resource management practices, may have an impact on the behavior and emotions of employees. Future research can conduct analyses from multiple perspectives.

## Data Availability Statement

The original contributions presented in the study are included in the article/supplementary material; further inquiries can be directed to the corresponding author.

## Ethics Statement

The studies involving human participants were reviewed and approved by Ethics Committee of Experimental Animals, South China University of Technology. The patients/participants provided their written informed consent to participate in this study.

## Author Contributions

SY and SL conceived and designed the work. XG collected the data. NW and SY analyzed and interpreted the data. XG and SL drafted the article. SY and NW are responsible for the modifications. All authors contributed to the article and approved the submitted version.

### Conflict of Interest

The authors declare that the research was conducted in the absence of any commercial or financial relationships that could be construed as a potential conflict of interest.
